# Dr. Lafayette Balcom

**Published:** 1905-10

**Authors:** 


DR.LAFAYETTE BALCOM, of Buffalo, a graduate of the Medical Department, University of Buffalo, 1864, died at Detroit, Mich., September 14, 1905, aged 67 years.

